# The effect of the pandemic on the intravitreal injection treatment for patients with age-related macular degeneration, regardless of lockdown periods

**DOI:** 10.22336/rjo.2025.27

**Published:** 2025

**Authors:** Mahmut Oğuz Ulusoy, Cansu Erseven, Büşra Erel, Sadık Görkem Çevik, İrfan Perente

**Affiliations:** 1 Department of Ophthalmology, Bursa Yüksek İhtisas Research and Training Hospital, Bursa, Turkey; 2 Department of Ophthalmology, Beyoğlu Research and Training Hospital, İstanbul, Turkey

**Keywords:** COVID-19, pandemic, intravitreal injection, age-related macular degeneration, COVID-19 = coronavirus disease 19, AMD = age related macular degeneration, CNV = choroidal neovascularization, VEGF = vascular endothelial growth factor, PRN = pro re nata, SD-OCT = spectral domain optical coherence tomgraphy, CMT = central macular thickness

## Abstract

**Purpose:**

To evaluate the impact of the pandemic on the intravitreal injection treatment routine for patients with neovascular age-related macular degeneration, regardless of lockdown periods.

**Methods:**

We evaluated the data of intravitreal injections between March 2020 and June 1, 2021, which was the last day of official restrictions. We divided into two groups: a regular treatment group and a group that was interrupted for at least 3 months without excuse. The initial visual acuity at the beginning of the pandemic, the last visual acuity, the visual acuity after interruption, the central macular thickness, and the interruption time were recorded.

**Results:**

A total of 156 patients’ data were evaluated. There are forty-eight patients in the regular treatment group, 77 in the interruption group, and 31 in the discontinued group. The visual loss was significantly higher in the interruption group (respectively, -0.11 ± 0.19 vs. 0.03 ± 0.16, p<0.001). Central macular thickness changes were higher in the interruption group (respectively, 31.8 ± 5.7µm vs. 13.5 ± 4.6, p<0.001). The mean interruption time was 6.71 ± 2.45 months.

**Discussion:**

Half of the patients in our study interrupted their treatment during the pandemic, and this interruption had a negative impact on their visual acuity and central macular thickness. In addition to COVID-19’s own high mortality and morbidity, it also had adverse effects on patients who required regular follow-up and treatment.

**Conclusion:**

Since interruptions in treatment negatively impact the prognosis of AMD, effective protocols that do not require frequent visits are necessary.

## Introduction

The novel coronavirus disease (COVID-19), caused by the severe acute respiratory syndrome coronavirus type 2 (SARS-CoV-2), primarily manifests as an acute upper and lower respiratory tract infection and can affect multiple organs and systems, including the heart, intestines, kidneys, blood, and nervous system. The disease’s primary mode of transmission is especially through droplets and direct human-to-human contact [1]. The pandemic resulted in numerous hospitalizations and deaths. The rapid and uncontrolled spread of the disease worldwide forced governments to lockdown cities, implement social distancing rules, cancel scientific meetings, and limit non-essential work [2]. Following the first COVID-19 case in Turkey, which was reported on March 11, 2020, the government imposed lockdowns on the country several times, and restriction periods were implemented.

Age-related macular degeneration (AMD) is a leading cause of progressive vision loss [3]. Neovascular age-related macular degeneration (nAMD) is a late-stage form, characterized by choroidal neovascularization (CNV). Inhibition of vascular endothelial growth factor (VEGF) with intravitreal anti-VEGF injection is effective in slowing down the angiogenic process. Three main treatment regimens are commonly employed: monthly injections, treat-and-extend, and pro re nata (PRN). The importance of frequent injections has been previously demonstrated in several studies for the long-term preservation of vision [3-9].

In this study, we aimed to investigate the effects of the COVID-19 pandemic on the treatment course of nAMD patients who received anti-VEGF injection therapy, independent of the restriction periods.

## Methods

In our study, we evaluated intravitreal injection records from March 2020 to June 1, 2021, encompassing the period from the beginning to the last day of the official restriction. Ethics committee approval (Number: 2011-KAEK-25 2022/11-16) was obtained from the Ethics Committee of Bursa Yüksek İhtisas Research and Training Hospital, Bursa, Turkey. The present study was conducted in accordance with the principles outlined in the Declaration of Helsinki.

We evaluated and compared the records of two groups of patients: those who regularly attended their treatment and those who interrupted therapy for at least 3 months, independent of the lockdown periods, without any apparent reason, and did not receive treatment in any center. These patients were then followed up again. The study included patients who had received at least three loading intravitreal anti-VEGF injections.

All patients underwent a detailed ophthalmic examination, including visual acuity (VA) testing using the Snellen chart, intraocular pressure measurement using non-contact tonometry, dilated fundus examination using 90D lens and central macular thickness (CMT) measurement using spectral domain optical coherence tomography (SD-OCT: RTVue XR AVANTI; AngioVue, Optovue Inc, Fremont, CA), at the beginning of the pandemic in both group and the last visit for the patients who came regularly and first visit after the break for interrupted group, were recorded. In addition to demographic data, the interruption period and macular scar development were recorded.

Patients with a history of ocular trauma, glaucoma, systemic hypertension, diabetes mellitus, or neurodegenerative disease were excluded from the study. In addition, to obtain clear images, patients with a refractive error of > ±6.00D were excluded from the study.

### Statistical Analysis

Statistical data were analysed using SPSS version 21.0 (SPSS, Chicago, IL, USA). Values were expressed as mean ± standard deviation. The normality of the values was analysed using the Kolmogorov-Smirnov test. Student t-test was used according to the Kolmogorov-Smirnov test results. Differences were considered significant at *p*<0.05.

## Results

A total of 156 patients’ data were analyzed. Of these patients, 48 (30.76%) continued treatment, 77 (49.35%) interrupted, and 31 (19.87%) discontinued treatment. None of the patients from the discontinued group returned to the clinic for follow-up or treatment. The mean age of the patients was 62.21 ± 6.74 years, and 56.4% (88/156) of the patients were female. There was no significant difference between the groups in terms of age and gender.

Vision loss was greater in the group that had an interruption compared to the group that continued the treatment (-0.11 ± 0.19 and 0.03 ± 0.16, respectively, p < 0.001). CMT change was found to be significantly higher in the interruption group compared to the other group (31.8 ± 5.7µm and 13.5 ± 4.6, respectively, p<0.001). Although macular scar development was more common in the interruption group, there was no significant difference (17/60 and 8/40, respectively; p = 0.46). The mean interval time was 6.71 ± 2.45 months (**Table 1**).

**Table 1:** Comparison of the demographic factors and results of the two groups

	Continued	Interruption	p
Age	61.8 ± 2.02	60.43 ± 1.92	0.68
Gender (M/F)	10/38	31/46	0.024
Eye (R/L)	16/32	40/37	0.042
Vo (change)	0.038 ± 0.16	-0.1 ± 0.19	<0.001
CMT (change)	13.5 ± 4.6	31.8 ± 5.7	<0.001
Interruption period (month)	-	6.71 ± 2.45	
Scar development (+/-)	8/40	17/60	0.46

CMT = central macular thickness

## Discussion

In this study, we evaluated the impact of the COVID-19 pandemic on intravitreal injection treatment, regardless of lockdown periods. We compared two groups: those who regularly attended appointments and those who interrupted treatment for more than 3 months without a valid reason. We found significant vision loss in the group who interrupted treatment compared to the regular group. In addition, CMT change and macular scar development were higher in the interruption group.

AMD is the primary cause of blindness in the elderly population of developed countries. nAMD is an advanced stage of this illness characterized by choroidal neovascularization. The primary purpose of treatment is to decrease the VEGF level through intravitreal injections [3]. Regular and appropriate injections are essential in the treatment period. The negative consequences of treatment interruption have been reported in numerous articles before the pandemic [10,11].

With the pandemic, the visit intervals of patients and the consequences of this led to prolonged intravitreal injection intervals [12-14]. The reduction in patient volume at clinics ranged from 25% to 81%, according to the analysis [15]. The discontinuation rate was reported to be 36.1% to 98.7% in a multicenter study. The visit rate decreased from a mean of 1.40 to 0.82 visits per month during the 6 months before lockdown to 0.12-0.24 visits per month during the lockdown, according to this study [14]. The attendance rate for appointments was found to be 42.3% in the restricted period in a clinic in Turkey [16]. A decrease in intravitreal injections and clinic visits was shown, especially in the AMD group, in another study. However, no difference was found in ROP injections [17]. Additionally, the injection interval was prolonged after the lockdown compared to the pre-lockdown period [12]. The mean duration of delay was found to be 4.12 ± 0.79 months in the nAMD group in a recent study [18]. Travel limitations, systemic comorbidities, and fear of COVID-19 were other factors contributing to discontinuation [19]. The most important cause of delay for ophthalmic situations was having better vision in their fellow eyes [15]. Teo KYC et al., who evaluated the widespread application of extended protocols during the pandemic, suggested that extensions in intravitreal injection intervals over 12 weeks should be avoided [20]. However, in another study, the authors were unable to observe any significant difference between the increase in treatment interval and the functional loss. They explained this result as the loading phase of Bevacizumab in patients with AMD, reducing long-term visual loss [21]. In a recent study comparing the visit counts of the pre-lockdown, lockdown, and post-lockdown periods, the authors reported a decrease during the lockdown period, followed by an increase after the lockdown. However, they observed that the patients with high-risk comorbidities did not return to intravitreal injections [22]. In our study, we found the delay period to be 6.71 ± 2.45 months, with a discontinuation rate of 19.87%. However, we did not evaluate the reason for this discontinuation among patients.

The effect of discontinuation of treatment during the pandemic period was evaluated in several studies, and the authors reported significant deterioration in vision [13,21,23]. In a recent study assessing the effect of the pandemic on various intravitreal injection-required diseases, it was reported that nAMD patients were more severely affected [24]. In another study, the authors built a mixed-effect model showing that the BCVA of patients decreased more than predicted after lockdown compared to central macular thickness [21]. Even if the intravitreal injections were restarted and anatomical recovery was achieved, no equivalent visual improvement was observed [13]. The reason for this non-parallel improvement was possible retinal cellular death, scar formation, and complications such as submacular hemorrhage. In a recent study, the authors suggested that BCVA decrease and subretinal scar formation are significantly greater in delayed polypoidal choroidal vasculopathy (PCV) and AMD patients [18]. However, in another study, they could not find a significant difference in the development of fibrosis between patients in the post-lockdown period and those who had a worse visual outcome [21]. In another study, the authors evaluated the results and reasons for discontinuation in European countries and found different delay intervals. However, their study was not designed to determine the reason for this difference; a typical result was observed in all countries, with a decrease in BCVA [23]. Similar to recent studies, we found that the interrupted group experienced significantly more vision loss than the other group, and macular scar formation was more prevalent in this group. However, it was not statistically significant.

## Conclusion

In conclusion, we evaluated the effect of treatment interruption, regardless of lockdown periods, during the pandemic. We demonstrated that, despite the absence of official restrictions, the pandemic’s spiral of fear affected the patients’ treatment periods. These results showed that we needed more effective and extended treatment protocols, and that this necessity might have arisen from various reasons, including the pandemic.
